# Acute kidney injury epidemiology, risk factors, and outcomes in critically ill patients 16–25 years of age treated in an adult intensive care unit

**DOI:** 10.1186/s13613-018-0373-y

**Published:** 2018-02-14

**Authors:** Dana Y. Fuhrman, Sandra Kane-Gill, Stuart L. Goldstein, Priyanka Priyanka, John A. Kellum

**Affiliations:** 1 Children’s Hospital of Pittsburgh, 4401 Penn Avenue, Children’s Hospital Drive, Faculty Pavilion Suite 2000, Pittsburgh, PA 15224 USA; 2 School of Pharmacy, University of Pittsburgh, 638 Salk Hall, 3501 Terrace Street, Pittsburgh, PA 15261 USA; 3 Center for Acute Care Nephrology, Cincinnati Children’s Hospital Medical Center, 3333 Burnet Avenue, Cincinnati, OH 45229 USA; 4 The Center for Critical Care Nephrology, 3347 Forbes Avenue, Ste 220, Pittsburgh, PA 15213 USA

**Keywords:** Young adult, Acute kidney injury (AKI), Critically ill

## Abstract

**Background:**

Most studies of acute kidney injury (AKI) have focused on older adults, and little is known about AKI in young adults (16–25 years) that are cared for in an adult intensive care unit (ICU). We analyzed data from a large single-center ICU database and defined AKI using the Kidney Disease Improving Global Outcomes criteria. We stratified patients 16–55 years of age into four age groups for comparison and used multivariable logistic regression to identify associations of potential susceptibilities and exposures with AKI and mortality.

**Results:**

AKI developed in 52.6% (*n* = 8270) of the entire cohort and in 39.8% of the young adult age group (16–25 years). The AUCs for the age categories were similar at 0.754, 0.769, 0.772, and 0.770 for the 16–25-, 26–35-, 36–45-, and 45–55-year age groups, respectively. For the youngest age group, diabetes (OR 1.89; 95% CI 1.09–3.29), surgical reason for admission (OR 1.79; 95% CI 1.44–2.23), severity of illness (OR 1.02; 95% CI 1.02–1.03), hypotension (OR 1.13; 95% CI 1.04–1.24), and certain medications (vancomycin and calcineurin inhibitors) were all independently associated with AKI. AKI was a significant predictor for longer length of stay, ICU mortality, and mortality after discharge.

**Conclusions:**

AKI is a common event for young adults admitted to an adult tertiary care center ICU with an associated increased length of stay and risk of mortality. Potentially modifiable risk factors for AKI including medications were identified for all stratified age groups.

## Background

An association of acute kidney injury (AKI) and adverse outcomes including length of hospital stay, progression to chronic kidney disease (CKD), and mortality is consistently shown in multiple patient populations [1–6]. In critically ill patients the rates of AKI vary based on the population studied and definition of AKI used, with reported rates of 8–89% for children [7–11] and 7–25% for adults [12–15]. Recently, investigators in the Assessment of Worldwide Acute Kidney Injury, Renal Angina, and Epidemiology (AWARE) study explored the association of AKI with morbidity and mortality in patients 3 months to 25 years of age admitted to a pediatric intensive care unit (ICU) [5]. However, no prior study has investigated specifically the incidence and implications of AKI in the young adult critically ill patient population treated in an adult ICU, a patient group that is growing in adult critical care practices [16].

Given the complexity and diversity of the critical care patient population, it can be challenging to identify and address the numerous risk factors for AKI encountered in the ICU. Since we continue to have no direct pharmacologic therapies for AKI, prevention is of paramount importance. An understanding of the potentially modifiable risk factors that may be unique to different patient groups within the ICU is critical to the prevention of AKI. As a result of an increasing number of individuals with childhood chronic illnesses surviving into adulthood [17], there is a need to understand the potentially unique modifiable risk factors for AKI in the 16–25-year-old or young adult ICU population. Little is known about the potential comorbid conditions that may exist in this age group, possibly impacting their AKI incidence and outcomes. As a result of certain comorbid conditions prior to entering the ICU, patients may have a greater exposure to nephrotoxic pharmacologic agents thereby potentially increasing their risk of AKI. Hui-Stickle et al. [18] demonstrated that nephrotoxic medications were the most common cause of acute renal failure for older children and adolescents, while ischemia was the most common etiology in patients 5 years of age or less. There have been no previous studies to date exploring the susceptibilities, exposures, and outcomes of AKI specifically in young adult ICU patients cared for outside of a children’s hospital. Thus, we sought to determine if the incidence, risks, and associated outcomes for AKI varied by age across a population of 16–55-year-old ICU patients treated in an adult hospital ICU.

## Methods

### Study population

**Fig. 1:**
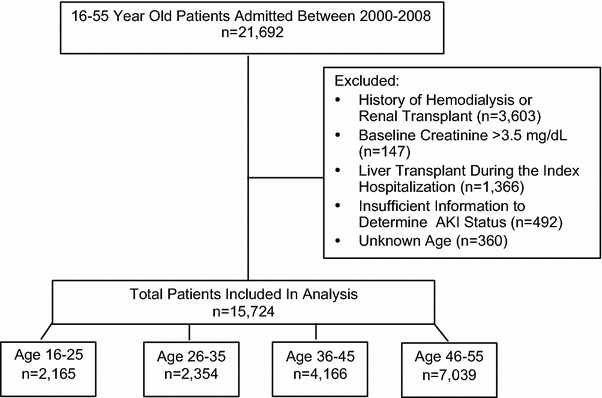
Flow diagram of the study cohort

After obtaining institutional review board approval, data were obtained from the High-Density Intensive Care (HiDenIC) database, which includes clinical variables on all patients admitted to the University of Pittsburgh, a tertiary care academic medical center, from July 2000–September 2008. The HiDenIC database includes data on adult patients admitted to one of eight ICUs (medical, cardiac, transplant, surgical, neurological, and trauma). Exclusion criteria were applied including: (1) history of hemodialysis or renal transplant, (2) baseline creatinine > 3.5 mg/dl, (3) liver transplant during the index hospitalization, (4) insufficient information to determine AKI status, and (5) unknown age (Fig. 1). We defined the young adult population as those individuals 16–25 years of age. The remaining cohort was stratified into 10-year age increments including: 26–35 years, 36–45 years, and 46–55 years.

### Clinical variables

The risk factors included the analysis are significant predictors of AKI in previous studies [19, 20]. The potential risk factors include sex, race, reference creatinine, estimated glomerular filtration rate (eGFR) derived from the reference creatinine [21], comorbid conditions defined by ICD-9 codes (cardiac disease, CKD, diabetes, fluid overload, history of hypertension, malignancies), admission type (medical or surgical), and moderate anemia (defined by The World Health Organization [22]). Fluid balance was calculated by subtracting total intake from output divided by the admission weight (kg) × 100 in the first 24 h of ICU admission [23]. We defined fluid overload as a fluid balance > 5%. Severity of illness was evaluated with the Acute Physiology and Chronic Healthy Evaluation (APACHE) III score [24]. In the first 24 h of ICU admission, the need for vasopressors, mechanical ventilation as well as concern for sepsis (the ordering of blood cultures and antibiotics within 24 h of each other) was also included. Additionally, we evaluated exposure to potentially nephrotoxic medications within the first 24 h of ICU admission, including angiotensin-converting enzyme inhibitors, angiotensin II receptor blockers, vancomycin, aminoglycosides, antibiotics other than vancomycin or aminoglycosides (including piperacillin/tazobactam, cephalosporins, quinolones, macrolides, sulfonamides, and carbapenems), calcineurin inhibitors, nonsteroidal anti-inflammatory drug (NSAID) medications, acyclovir, mannitol, and phenytoin.

### Outcomes

We defined AKI according to the Kidney Disease Improving Global Outcomes (KDIGO) criteria [4]. Any patient meeting the criteria for KDIGO stage 1 or more based on either serum creatinine or urine output during their ICU stay was deemed to have AKI. We defined the reference creatinine as the baseline creatinine when available (lowest value between the most recent hospital creatinine value up to 1 year prior the index hospital admission and the creatinine recorded in the first 24 h of hospital admission) or the lowest value between the creatinine recorded in the first 24 h of hospital admission, first 24 h of ICU admission, and (for patients without a history of CKD) the creatinine derived from the Modification of Diet in Renal Disease (MDRD) equation for creatinine using an eGFR of 75 ml/min/1.73 m^2^ [25, 26]. The reference creatinine was used to determine creatinine changes for defining AKI. We evaluated for each age strata rates of AKI, need for renal replacement therapy (RRT), recovery from RRT, ICU length of stay, hospital length of stay, ICU mortality, hospital mortality, 90-day mortality, and 1-year mortality.

### Statistical analysis

Categorical variables were summarized as number and percentage, and continuous variables were summarized as median with interquartile range. Given the large number of patients in the study, statistical differences alone are unlikely to be meaningful. Therefore, we set 10% as a clinically meaningful difference between age groups. Age per 5 years was included as a risk factor for each age group to account for differences within the age groups. To determine the susceptibilities and exposures associated with AKI, multivariable logistic regression was performed whereby: (1) the cohort was stratified by age group (each of 10 years, starting from age 16–25) and (2) with age group as a main effect and accounting for interactions between age group and all other risk factors. Age-stratified models were built using the following steps: (1) adding each risk factor to age as a continuous variable and using the Wald statistic to determine their significance, (2) the individual size of all variables in step 1 was tested with the Wald statistic as they were added to a multivariable logistic regression model, (3) variables with *p* ≥ 0.05 were taken out of the model and a reduced model was fit, and (4) lastly to compare nested models in steps 2 and 3 the likelihood ratio test was used to determine a final model. For the interaction models, in order to find a main effects model, age group was used as a main effect and steps 1 through 4 were repeated. With age retained in the models regardless of significance level, all possible interactions were added one at a time and their significance was determined with the Wald statistic. STATA’s “roctab” function was used to assess the area under the receiver operating characteristic curve (AUC) for each age-stratified model. In addition, the “rocreg” function that uses bootstrap (1000 replications) for inference was also used to assess nonparametric ROC estimation under the presence of covariates. Model selection for ICU mortality, hospital mortality, mortality at 90 days after ICU admission, and mortality 1 year after ICU admission across age groups was done using the stepwise selection methodology described above to identify the best model for mortality prediction. Goodness of fit was assessed using Hosmer–Lemeshow [27]. Statistical analyses were performed using STATA software (version SE 14.0, StataCorp LP) and SAS 9.4 with statistical significance set at *p* < 0.05.

## Results

After applying the exclusion criteria, 15,724 patients were included in the analysis. The reference creatinine was determined from a documented baseline creatinine in 5543 patients and estimated in 10,181 patients. AKI occurred in 8270 (52.6%) patients. The characteristics of individuals that developed AKI are shown in Table 1. In the 16–25-year-old age group, 39.8% of the patients developed AKI. In all age groups stage I AKI occurred with the greatest frequency. Although stage 3 AKI occurred with the lowest frequency in the 16–25-year-old age group, it occurred in 15% of patients. Only a few variables met our criteria of 10% as a clinically meaningful difference between age groups. Cardiac disease, hypertension, multiple comorbidities, vasopressor use and NSAID use were more common in older adults (Table 1). The distributions for the remainder of the variables were similar between groups.

**Table 1:** Patient characteristics by age group (years) with acute kidney injury

Characteristic	16–25	26–35	36–45	46–55	All
	*N* = 862	*N* = 1098	*N* = 2189	*N* = 4121	*N* = 8270
% of age group with AKI	39.8	46.6	52.5	58.5	52.6
Age (years), Median (*Q*1–*Q*3)	22 (19–24)	31 (28–33)	41 (39–44)	51 (48–53)	45 (36–51)
Males *N* (%)	573 (66.5)	670 (61.2)	1304 (60)	2500 (61)	5047 (61)
Race *N* (%)					
White	597 (69)	790 (72)	1654 (76)	3127 (76)	6168 (76)
Black	132 (15)	131 (12)	226 (10)	409 (10)	898 (11)
Other	133 (15)	177 (16)	309 (14)	585 (14)	1204 (15)
Reference creatinine (mg/dl), median (*Q*1–*Q*3)	0.9 (0.7–1.1)	0.9 (0.7–1.1)	0.9 (0.7–1.1)	0.9 (0.7–1.1)	0.9 (0.7–1.1)
eGFR (ml/min/1.73 m^2^), median (*Q*1–*Q*3)	121.2 (92.8–133)	111.4 (81.9–123.1)	95.7 (79.4–113.9)	86.2 (76.9–104.8)	96.5 (78.6–113.2)
Fluid balance > 5%	111 (12.9)	161 (14.7)	285 (13.1)	485 (11.8)	1042 (12.6)
Cardiac disease *N* (%)	41 (5)	95 (9)	225 (10)	666 (16)	1027 (12)
Chronic kidney disease *N* (%)	16 (2)	26 (2)	135 (6)	218 (5)	395 (5)
Diabetes *N* (%)	50 (6)	102 (9)	227 (10)	741 (18)	1120 (14)
History of hypertension *N* (%)	59 (7)	135 (12)	459 (21)	1274 (31)	1927 (23)
Malignancy *N* (%)	7 (0.8)	15 (1)	62 (2.8)	156 (3.8)	240 (3)
Multiple comorbidities *N* (%)	142 (17)	290 (26)	803 (37)	1889 (46)	3124 (38)
Mechanical ventilation *N* (%)	580 (67)	684 (62)	1340 (61)	2413 (59)	5017 (61)
Surgical admission *N* (%)	504 (65)	601 (60)	1128 (57)	2216 (59)	4449 (59)
Suspected sepsis *N* (%)	122 (14)	198 (18)	395 (18)	698 (17)	1413 (17)
APACHE III score, median (*Q*1–*Q*3)	54 (36–72)	53 (34–73)	52 (35–74)	57 (38–80)	55 (37–76)
Vasopressor use *N* (%)	149 (17)	224 (20)	521 (24)	1125 (27)	2019 (24)
Moderate anemia *N* (%)	227 (26)	315 (29)	577 (26)	1259 (31)	2378 (29)
Maximum KDIGO *N* (%)					
Stage 1	400 (46)	444 (41)	738 (34)	1241 (30)	2823 (34)
Stage 2	335 (39)	433 (39)	906 (41)	1783 (43)	3457 (42)
Stage 3	127 (15)	221 (20)	545 (25)	1097 (27)	1990 (24)
Medication exposure *N* (%)					
ACE inhibitor/ARB	21(2)	27 (2)	93 (4)	269 (7)	410 (5)
Vancomycin	124 (14)	203 (18)	373 (17)	669 (16)	1369 (17)
Aminoglycoside	31 (3.6)	44 (4)	87 (4)	157 (4)	319 (4)
Other Antibiotics	62 (7)	82 (7)	137 (6)	258 (6)	539 (6)
Calcineurin inhibitor	58 (7)	94 (9)	153 (7)	264 (6)	569 (7)
NSAID	40 (5)	90 (8)	269 (12)	716 (17)	1115 (14)
Acyclovir	18 (2)	29 (2.6)	462 (2.1)	67 (1.6)	160 (2)
Mannitol	28 (3)	26 (2)	32 (1.5)	30 (0.7)	116 (1)
Phenytoin	32 (3.7)	24 (2)	47 (2)	82 (2)	185 (2)

*AKI* acute kidney injury, *eGFR* estimated glomerular filtration rate, *APACHE* Acute Physiology and Chronic Healthy Evaluation, *KDIGO* Kidney Disease Improving Global Outcomes, *ACE* angiotensin-converting enzyme, *ARB* angiotensin II receptor blocker, *NSAID* nonsteroidal anti-inflammatory drug

Sepsis and vancomycin use were found to be highly associated with AKI in the overall cohort as well as for each individual age group. Given that sepsis was defined as the ordering of blood cultures and antibiotics within 24 h of each other and vancomycin was the most common antibiotic prescribed, not surprisingly sepsis was highly colinear with vancomycin. Therefore, sepsis was not included in the final individual logistic regression models built for each age group (Table 2). The area under the curve (AUC) for each of the four age groups was similar indicating a comparable ability to predict AKI across the different age strata at 0.754, 0.769, 0.772, and 0.770 for the 16–25-, 26–35-, 36–45-, and 46–55-year-old age groups, respectively. In order to gain more precise estimates, the AUC was re-fitted using bootstrapping and similar AUC values were also determined across the four age strata. Diabetes, APACHE III score, and vancomycin were significantly positively associated with AKI across all age groups. Specifically, for the young adults (ages 16–25), age, race, diabetes, surgical admission, APACHE III score, hypotensive index, vancomycin, calcineurin inhibitor, NSAID, and other nephrotoxic medication use were all significantly associated with AKI (Table 2). Included in the category of other nephrotoxic medications were acyclovir, mannitol, and phenytoin. However, when each of these drugs was included individually in the model, there was no significant association with AKI.

**Table 2:** Multivariable logistic regression of risk factors for individuals with acute kidney injury compared to those without acute kidney injury by age categories (years)

Characteristic	16–25 OR (95% CI *p* value)	26–35 OR (95% CI *p* value)	36–45 OR (95% CI *p* value)	46–55 OR (95% CI *p* value)
Age per 5 years	1.39 (1.14–1.69, < 0.01)	1.21 (1.02–1.43, 0.02)	–	–
Black	1.41 (1.04–1.91, 0.02)	–	–	–
Other race	–	–	–	–
Diabetes	1.89 (1.09–3.29, 0.02)	1.86 (1.20–2.89, < 0.01)	1.52 (1.14–2.02, 0.01)	1.55 (1.28–1.85, 0.01)
Fluid balance > 5%	–	–	–	–
Malignancy	–	–	–	–
Hypertension	–	–	–	–
Cardiac disease	–	3.75 (2.23–6.29, < 0.01)	2.00 (1.47–2.72, < 0.01)	1.36 (1.12–1.64, 0.01)
Chronic kidney disease	–	–	–	–
Surgical admission	1.79 (1.44–2.23, < 0.01)	1.39 (1.14–1.71, < 0.01)	1.23 (1.05–1.72, < 0.01)	–
Vasopressor use	–	1.48 (1.04–2.12, 0.03)	1.34 (1.05–1.65, 0.01)	1.41 (1.19–1.69, < 0.01)
Mechanical ventilation	–	1.39 (1.09–2.12, < 0.01)	1.38 (1.16–1.31, < 0.01)	1.53 (1.33–1.73, < 0.01)
Moderate anemia	–	–	–	–
APACHE III score	1.02 (1.02–1.03, < 0.01)	1.02 (1.02–1.26, < 0.01)	1.03 (1.02–1.04, < 0.01)	1.03 (1.03–1.03, < 0.01)
eGFR	–	–	0.98 (0.98–0.99, < 0.01)	0.98 (0.98–0.99, < 0.01)
Hypotensive Index	1.13 (1.04–1.24, < 0.01)	–	–	1.04 (1.01–1.07, 0.01)
ACE inhibitor/ARB	–	–	–	–
Vancomycin	1.46 (1.00–2.13, 0.04)	1.56 (1.13–1.39, < 0.01)	1.39 (1.08–1.77, 0.01)	1.45 (1.18–1.77, < 0.01)
Other antibiotics	–	–	–	–
Calcineurin inhibitor	2.72 (1.45–5.12, < 0.01)	–	2.45 (1.59–3.75, < 0.01)	–
NSAID	0.51 (0.32–0.82, < 0.01)	0.68 (0.49–0.80, 0.02)	0.78 (0.63–0.96, 0.01)	0.92 (0.80–1.07, 0.03)
Other nephrotoxic medications	1.60 (1.03–2.49, 0.03)	–		–
AUC (*Q*1–*Q*3)	0.754 (0.732–0.776)	0.769 (0.749–0.789)	0.772 (0.757–0.787)	0.770 (0.758–0.781)

*OR* odds ratio, *CI* confidence interval, *APACHE* Acute Physiology and Chronic Healthy Evaluation*, eGFR* estimated glomerular filtration rate, *ACE* angiotensin-converting enzyme, *ARB* angiotensin II receptor blocker, *NSAID* nonsteroidal anti-inflammatory drug, *ROC* receiver operator curve

Statistically significant interactions between age groups and the potential risk factors were determined (Table 3). Despite a similar ability to predict AKI across the four age strata, certain risk factors were significantly different with respect to age group. The risk factors that had significant interactions with age were cardiac disease, surgical admission, eGFR, calcineurin inhibitor, NSAID, and other nephrotoxic medication use.

**Table 3:** Multivariable logistic regression of interactions between age groups and risk factors associated with AKI

Interaction of age group with	*χ*^2^ (*df*)	*p* value*
Race	5.19 (6)	0.52
Diabetes	4.31 (3)	0.22
Cardiac disease	17.55 (3)	< 0.01
Chronic kidney disease	4.67 (3)	0.19
Surgical admission	18.42 (3)	< 0.01
Vasopressor use	1.73 (3)	0.62
Mechanical ventilation	4.57 (3)	0.20
Moderate anemia	2.02 (3)	0.56
APACHE III score	7.07 (3)	0.06
eGFR	16.5 (3)	< 0.01
Hypotensive Index	2.25 (3)	0.52
Vancomycin	1.06 (3)	0.78
Calcineurin inhibitor	12.84 (3)	< 0.01
NSAID	3.30 (3)	< 0.01
Other nephrotoxic medications	8.08 (3)	0.04

*AKI* acute kidney injury, *APACHE* Acute Physiology and Chronic Healthy Evaluation*, eGFR* estimated glomerular filtration rate, *NSAID* nonsteroidal anti-inflammatory drug

*Each *p* value comes from a different multivariable logistic regression with age group, 15 risk factors and one interaction

**Fig. 2:**
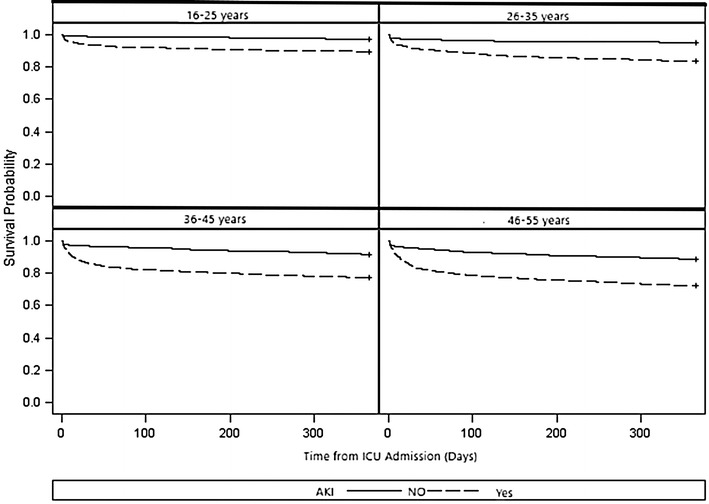
Kaplan–Meier survival curves for 1-year mortality after ICU discharge

Table 4 shows outcomes of the patients with AKI in each of the four age strata. In the young adult patients, even though only a small number of patients received RRT (*n* = 46), 47.8% of patients that received RRT while hospitalized had no recovery from RRT at 90 days. In the 640 patients in the overall patient cohort that received RRT, 59.4% had no recovery from RRT at 90 days. The ICU and hospital length of stay were similar between age groups. Hospital, ICU, 90-day, and 1-year mortality were greater in the older adult groups. AKI was a significant predictor of hospital mortality, ICU mortality, mortality at 90 days and mortality at 1 year in the young adult patients (Table 5). Tables 6 and 7 show the significant role that AKI contributed toward predicting hospital and 1 year post-discharge mortality in the 16–25-year-old age group. Patients with AKI had an increased risk of 1 year post-discharge mortality in all age groups (Fig. 2). Among the variables included in the multivariable logistic regression, it was the APACHE III score, a diagnosis of malignancy, and a diagnosis of AKI during the time of ICU admission only that significantly contributed toward predicting mortality 1 year after discharge (Table 7).

**Table 4:** Outcomes of patients with acute kidney injury for each of the four age categories (years)

Outcome	Age 16–25	Age 26–35	Age 36–45	Age 46–55	All
Need for RRT *N* (%)	46 (5.3)	77 (7)	172 (7.9)	345 (8.4)	640 (7.7)
No recovery from RRT at 90 Days *N* (%)	22 (47.8)	37 (48.1)	92 (53.5)	229 (66.4)	380 (59.4)
ICU length of stay (days), mean (SD)	9.6 (10.7)	10.3 (15.1)	8.7 (11.6)	8.8 (12.9)	9 (12.7)
Hospital length of stay (days), mean (SD)	20.1 (22.2)	21.7 (25.4)	19.5 (24.1)	19.5 (24.2)	19.9 (24.1)
ICU mortality *N* (%)	51 (5.9)	80 (7.3)	245 (11.2)	486 (11.8)	862 (10.4)
Hospital mortality *N* (%)	64 (7.4)	107 (9.7)	319 (14.6)	674 (16.4)	1164 (14.1)
90-Day mortality *N* (%)	67 (7.8)	123 (11.2)	390 (17.8)	860 (20.9)	1440 (17.4)
1-Year mortality *N* (%)	90 (10.4)	180 (16.4)	498 (22.8)	1145 (27.8)	1913 (23.1)

*RRT* renal replacement therapy, *ICU* intensive care unit, *SD* standard deviation

**Table 5:** Multivariable logistic regression of outcomes related to acute kidney injury by age categories (years)

Outcome	Age 16–25 OR (95% CI *p* value)	Age 26–35 OR (95% CI *p* value)	Age 36–45 OR (95% CI *p* value)	Age 46–55 OR (95% CI *p* value)	All OR (95% CI *p* value)
Hospital mortality	2.48 (1.25–4.90, < 0.01)	8.63 (1.04–71.70, 0.04)	21.73 (4.02–117.44, < 0.01)	4.88 (2.55–9.34, < 0.01)	2.03 (1.69–2.43, < 0.01)
ICU mortality	2.78 (1.30–5.94, < 0.01)	1.024 (0.58–1.82, 0.934)	1.67 (1.11–2.51, 0.01)	1.48 (1.12–1.97, 0.07)	3.74 (2.09–6.68, < 0.01
Mortality at 90 days	2.04 (1.09–3.82, 0.03)	1.313 (0.84–2.06, 0.233)	2.31 (1.70–3.14, < 0.01)	1.63 (1.34–1.98, < 0.01)	1.78 (1.53–2.07, < 0.01)
Mortality at 1 year	2.25 (1.14–4.45, 0.02)	2.50 (1.50–4.15, < 0.01)	1.98 (1.45–2.70, < 0.01)	1.90 (1.54–2.36, < 0.01)	2.03 (1.74–2.39, < 0.01)

*OR* odds ratio, *CI* confidence interval, *ICU* Intensive Care Unit

**Table 6:** Multivariable logistic regression of risk factors for hospital mortality by age categories (years)

Outcome	Age 16–25 OR (95% CI *p* value)	Age 26–35 OR (95% CI *p* value)	Age 36–45 OR (95% CI *p* value)	Age 46–55 OR (95% CI *p* value)	All OR (95% CI *p* value)
Age per 5 years	0.73 (0.43–1.23, 0.23)	1.26 (0.88–1.80, 0.20)	1.51 (1.19–1.93, < 0.01)	1.23 (1.04–1.44, 0.01)	1.14 (1.09–1.18, < 0.01)
AKI	2.48 (1.25–4.90, < 0.01)	1.65 (0.98–2.79, 0.05)	2.06 (1.44–2.95, < 0.01)	2.00 (1.56–2.56, < 0.01)	2.03 (1.69–2.43, < 0.01)
APACHE III score	1.03 (1.01–1.03, < 0.01)	1.03 (1.02–1.03, < 0.01)	1.03 (1.02–1.03, < 0.01)	1.03 (1.02–1.03, < 0.01)	1.03 (1.03–1.04, < 0.01
Hypotensive Index	1.01 (1.01–1.02, < 0.01)	1.00 (0.99–1.01, 0.12)	1.00 (1.00–1.01, < 0.01)	1.00 (1.00–1.00, < 0.01)	1.01 (1.01–1.01, < 0.01)
Vasopressors	3.28 (1.60–6.75, < 0.01)	3.98 (2.46–6.43, < 0.01)	2.46 (1.79–3.38, < 0.01)	1.97 (1.61–2.42, < 0.01)	2.30 (2.0–2.7, < 0.01)
Surgical Admission	0.63 (0.35–1.15, < 0.01)	0.66 (0.43–1.01, 0.05)	0.54 (0.41–0.71, < 0.01)	0.60 (0.49–0.72, < 0.01)	0.60 (0.51–0.68, < 0.01)
Moderate anemia	1.12 (0.60–2.11, 0.72)	0.81 (0.51–1.3, 0.39)	0.87 (0.64–1.19, 0.41)	0.86 (0.70–1.05, 0.14)	0.87 (0.75–1.01, 0.07)
Hypertension	0.76 (0.21–2.69, 0.67)	0.85 (0.42–1.72, 0.65)	0.89 (0.61–1.30, 0.57)	0.94 (0.74–1.18, 0.59)	0.89 (0.74–1.07, 0.20)
Malignancy	5.23 (1.47–18.61, 0.01)	2.55 (1.09–5.94, 0.02)	1.80 (1.10–2.96, 0.01)	1.52 (1.12–2.05, 0.00)	1.68 (1.32–2.14, < 0.01)
Chronic liver disease	0.61 (0.06–6.01, 0.67)	1.37 (0.57–3.25, 0.47)	1.49 (0.93–2.4, 0.09)	1.56 (1.17–2.08, 0.00)	1.47 (1.16–1.85, < 0.01)
Multiple comorbidities	1.27 (0.51–3.17, 0.60)	1.58 (0.87–2.88, 0.13)	1.18 (0.80–1.73, 0.38)	0.82 (0.63–1.06, 0.13)	1.02 (0.84–1.24, 0.84)
AUC (*Q*1–*Q*3)	0.895 (0.854–0.932)	0.8942 (0.868–0.920)	0.8855 (0.868–0.902)	0.853 (0.838–0.867)	0.875 (0.865–0.884)

*RRT* renal replacement therapy, *ICU* intensive care unit, *SD* standard deviation

**Table 7:** Multivariable logistic regression of risk factors for 1-year mortality by age categories (years)

Outcome	Age 16–25 OR (95% CI *p* value)	Age 26–35 OR (95% CI *p* value)	Age 36–45 OR (95% CI *p* value)	Age 46–55 OR (95% CI *p* value)	All OR (95% CI *p* value)
Age	1.04 (0.94–1.16, 0.47)	1.03 (0.96–1.10, 0.34)	1.04 (0.99–1.09, 0.06)	1.04 (1.01–1.07, 0.01)	1.04 (1.03–1.04, < 0.01)
AKI	2.25 (1.14–4.45, 0.02)	2.50 (1.50–4.15, < 0.01)	1.98 (1.45–2.70, < 0.01)	1.90 (1.54–2.36, < 0.01)	2.03 (1.74–2.39, < 0.01)
Race^a^	0.63 (0.23–1.73, 0.37)	0.53 (0.25–1.11, 0.09)	0.62 (0.39–0.99, 0.05)	0.745 (0.55–1.02, 0.07)	0.67 (0.53–0.84, < 0.01)
APACHE III score	1.02 (1.01–1.03, < 0.01)	1.02 (1.02–1.03, < 0.01)	1.02 (1.02–1.03, < 0.01)	1.02 (1.02–1.03, < 0.01)	2.04 (1.73 –2.39, < 0.01)
BMI	0.98 (0.94–1.01, 0.20)	1.01 (0.99–1.03, 0.56)	0.99 (0.98–1.01, 0.46)	0.99 (0.98–0.99, 0.02)	0.99 (0.99–0.99, 0.02)
Hypotensive Index	1.01 (1.00–1.02, 0.06)	1.00 (0.99–1.01, 0.44)	1.01 (1.00–1.01, < 0.01)	1.00 (1.00–1.01, 0.01)	1.00 (1.00–1.01, < 0.01)
Vasopressors	1.99 (0.94–4.20, 0.07)	2.41 (1.54 –3.81, < 0.01)	1.62 (1.20–2.18, < 0.01)	1.35 (1.11–1.64, < 0.01)	1.54 (1.33–1.80, < 0.01)
Surgical admission	0.80 (0.44–1.46, 0.47)	0.52 (0.36–0.77, < 0.01)	0.53 (0.42– 0.69, < 0.01)	0.52 (0.44–0.62, < 0.01)	0.54 (0.47–0.61, < 0.01)
Hypertension	1.17 (0.42–3.27, 0.77)	0.80 (0.42–1.52, 0.50)	0.85 (0.61–1.19, 0.35)	0.85 (0.69–1.05, 0.13)	0.82 (0.69–0.97, 0.02)
Malignancy	17.84 (4.66–68.35, < 0.01)	2.43 (1.08–5.45, 0.03)	1.08 (0.62–1.88, 0.08)	2.04 (1.48–2.80, < 0.01)	1.91 (1.49–2.46, < 0.01)
Chronic liver disease	2.65 (0.77 –8.92, 0.12)	0.90 (0.43–1.88, 0.76)	1.26 (0.83 –1.92, 0.27)	1.70 (1.30–2.20, < 0.01)	1.48 (1.21–1.82, < 0.01)
History of COPD	13.96 (0.73– 268.49, 0.08)	1.31 (0.40–4.46, 0.66)	1.21 (0.68 –2.17, 0.52)	1.41 (1.06–1.89, 0.02)	1.30 (1.01–1.68, 0.04)
Multiple comorbidities	2.00 (0.87–4.61, 0.10)	3.22 (1.88–5.54, < 0.01)	2.07 (1.45 –2.95, < 0.01)	1.06 (0.84–1.36, 0.61)	1.53 (1.28–1.83, < 0.01)
AUC (*Q*1–*Q*3)	0.862 (0.816–0.907)	0.848 (0.818–0.878)	0.816 (0.794–0.837)	0.799 (0.783–0.814)	0.823 (0.812–0.834)

*AKI* acute kidney injury, *APACHE* Acute Physiology and Chronic Healthy Evaluation, *BMI* Body Mass Index, *COPD* chronic obstructive pulmonary disease

^a^Black compared to white

## Discussion

Even in young adult patients, AKI occurred in 39.8%. Similar to the AWARE study, a strength of this investigation is that we defined AKI using both the KDIGO serum creatinine and urine output criteria, given that defining AKI using serum creatinine has been shown to decrease the sensitivity for AKI detection [5]. Our rate of AKI in the young adult patient cohort falls between the rates reported by AWARE (26.9%) and the Acute Kidney Injury-Epidemiologic Prospective Investigation (AKI-EPI) published in 2015 (57.3%) [5, 28].

Importantly, we show AKI to be a significant predictor of hospital and ICU mortality as well as mortality after discharge in young adult critically ill patients, treated in an adult ICU. Similarly, including both ICU and non-ICU patients less than 18 years of age, Sutherland et al. show a significant association of AKI (defined using the KDIGO criteria) with ICU mortality and hospital length of stay. However, in contrast to our findings, they describe no significant association of AKI with mortality outside of the ICU [6]. Along with differences in patient age, the inclusion of patients with non-ICU AKI in their study may explain the discrepant results when compared to ours, which show AKI to be a significant predictor of hospital and 1 year post-discharge mortality in the 16–25-year-old age group.

Given the poor outcomes associated with AKI in this study as well as prior studies, it is imperative that those caring for critically ill patients identify and address the risk factors that are unique to specific patient groups. The results of this analysis suggest that there are potentially modifiable risk factors for AKI in critically ill young adults. The use of medications such as vancomycin and calcineurin inhibitors was found to be significantly associated with AKI in patients 16–25 years of age. When grouped together, mannitol, acyclovir, and phenytoin were uniquely associated with AKI in the 16–25-year-old cohort. Due to the low rate of use of these medications in our overall patient cohort, we cannot make any determination about AKI risk of each medication individually. The significant association of nephrotoxic medications and AKI in the 16–25-year-old age group suggests the need for future studies exploring the effect of different drug combinations in young adults on AKI development. At the bedside, frequent evaluations for transitioning medications to less nephrotoxic alternatives along with the use of therapeutic drug monitoring when available should be initiated.

The multivariable logistic regression demonstrates a “protective effect” of NSAID use on AKI risk across all of the age groups. NSAIDs are frequently withheld from patients in the ICU due to the concern of nephrotoxicity [29]. We speculate that our study results are due to a possible healthy user bias whereby patients without CKD and/or less comorbid conditions may have been more likely to receive NSAIDs during their ICU stay than patients with an elevated creatinine values and more comorbidities. However, given that increased NSAID use was not found to be positively associated with AKI in this critically ill patient cohort, intermittent NSAID use in patients without underlying renal disease who are euvolemic should be considered for analgesia given the low risk of renal side effects, as discussed in previous investigations [30, 31]. This may be particular important given recent attention to opiate use in the critically ill.

In this study, which did not include patients over 55 years of age, we show a similar ability to predict AKI when comparing the four age strata. Also using the HiDenIC database, but including ICU patients 55 years and older, Kane-Gill et al. [20] demonstrated that the ability of similar variables used in our study to predict AKI decreases with age. Specifically, they report that for patients greater than or equal to 75 years of age an AUC for predicting AKI of 0.673 [20]. This is in contrast to the higher AUC values determined in this study for younger adults, which demonstrates the superior ability to predict and, therefore, potentially prevent AKI in patients less than 55 years of age. Notably, the risk factors between those age 16–55 in our study and those > 75 in the previously published study varied with different drugs, history of hypertension, and sepsis in the older adult group [20].

Our models for mortality were also quite robust with all AUCs of at least 0.80 and some approaching 0.90 (Tables 6, 7). While our intent was not to develop a risk prediction model for mortality with AKI, and our models are likely overtrained, these results are far better than most reports in the literature [32]. Use of younger patients and stratification by age group may have led to significantly better predicative value. While the AUC values appear to increase with decreasing age, the confidence intervals overlap. Future studies are needed to validate these models in independent populations.

Our study has important limitations. The identified risk factors for AKI may be surrogates of other variables. For example, the association of calcineurin inhibitor use and AKI may reflect the association of AKI with transplant status. Notably, surgical as opposed to medical admission was an increasingly powerful risk factor for AKI in younger patients, especially those 16–25 years of age. However, it could not be determined from the database if surgeries were elective versus emergent. APACHE III scores and the MDRD equation for estimating GFR have not been validated in patients less than 18 years of age. Importantly, the MDRD equation was derived from patients with CKD, and its use in patients with critical illness is unclear. All of the young adults in this study were treated in adult intensive care units at a single institution. Given the single-center nature of this study, the comorbidities of a young adult patient group treated at other institutions may be different from our cohort of patients. Therefore, the results should be validated at other centers.

## Conclusions

Using the KDIGO criteria for both serum creatinine and urine output to define AKI, 39.8% of patients between the ages of 16–25 met AKI criteria during admission to an adult tertiary care center, indicating that AKI is a common event in this patient group. The diagnosis of AKI during hospital admission independently contributed toward increased hospital mortality, increased ICU mortality and increased mortality 90 days and 1 year after hospital discharge in the young adult patients. Potentially modifiable risk factors for AKI were identified, most notably nephrotoxic medication exposure. Risk factors identified in this younger population varied from published data in older adults (> 75 years old).

## Abbreviations

AKI: acute kidney injury
CKD: chronic kidney disease
AWARE: Assessment of Worldwide Acute Kidney Injury, Renal Angina, and Epidemiology
ICU: intensive care unit
HiDenIC: High-Density Intensive Care
eGFR: estimated glomerular filtration rate
APACHE: Acute Physiology and Chronic Health Evaluation
NSAID: nonsteroidal anti-inflammatory drug
KDIGO: Kidney Disease Improving Global Outcomes

## References

[1] Uchino S, Kellum JA, Bellomo R (2005). Acute renal failure in critically ill patients: a multinational, multicenter study. JAMA.

[2] Liano F, Pascual J (1996). Epidemiology of acute renal failure: a prospective, multicenter, community-based study. Madrid Acute Renal Failure Study Group. Kidney Int.

[3] Ali T, Khan I, Simpson W (2007). Incidence and outcomes in acute kidney injury: a comprehensive population-based study. J Am Soc Nephrol.

[4] Kidney Disease: Improving Global Outcomes (KDIGO) Work Group (2012). KDIGO clinical practice guildeline for acute kidney injury. Kidney Int Suppl.

[5] Kaddourah A, Basu RK, Bagshaw SM, Goldstein SL, Investigators A (2017). Epidemiology of acute kidney injury in critically ill children and young adults. N Engl J Med.

[6] Sutherland SM, Byrnes JJ, Kothari M (2015). AKI in hospitalized children: comparing the pRIFLE, AKIN, and KDIGO definitions. Clin J Am Soc Nephrol.

[7] Bailey D, Phan V, Litalien C (2007). Risk factors of acute renal failure in critically ill children: a prospective descriptive epidemiological study. Pediatr Crit Care Med.

[8] Schneider J, Khemani R, Grushkin C, Bart R (2010). Serum creatinine as stratified in the RIFLE score for acute kidney injury is associated with mortality and length of stay for children in the pediatric intensive care unit. Crit Care Med.

[9] Zappitelli M, Parikh CR, Akcan-Arikan A, Washburn KK, Moffett BS, Goldstein SL (2008). Ascertainment and epidemiology of acute kidney injury varies with definition interpretation. Clin J Am Soc Nephrol.

[10] Akcan-Arikan A, Zappitelli M, Loftis LL, Washburn KK, Jefferson LS, Goldstein SL (2007). Modified RIFLE criteria in critically ill children with acute kidney injury. Kidney Int.

[11] Basu RK, Zappitelli M, Brunner L (2014). Derivation and validation of the renal angina index to improve the prediction of acute kidney injury in critically ill children. Kidney Int.

[12] Groeneveld AB, Tran DD, van der Meulen J, Nauta JJ, Thijs LG (1991). Acute renal failure in the medical intensive care unit: predisposing, complicating factors and outcome. Nephron.

[13] de Mendonca A, Vincent JL, Suter PM (2000). Acute renal failure in the ICU: risk factors and outcome evaluated by the SOFA score. Intensive Care Med.

[14] Brivet FG, Kleinknecht DJ, Loirat P, Landais PJ (1996). Acute renal failure in intensive care units–causes, outcome, and prognostic factors of hospital mortality; a prospective, multicenter study. French Study Group on Acute Renal Failure. Crit Care Med.

[15] Wilkins RG, Faragher EB (1983). Acute renal failure in an intensive care unit: incidence, prediction and outcome. Anaesthesia.

[16] Edwards JD, Houtrow AJ, Vasilevskis EE, Dudley RA, Okumura MJ (2013). Multi-institutional profile of adults admitted to pediatric intensive care units. JAMA Pediatr.

[17] Janse AJ, Uiterwaal CS, Gemke RJ, Kimpen JL, Sinnema G (2005). A difference in perception of quality of life in chronically ill children was found between parents and pediatricians. J Clin Epidemiol.

[18] Hui-Stickle S, Brewer ED, Goldstein SL (2005). Pediatric ARF epidemiology at a tertiary care center from 1999 to 2001. Am J Kidney Dis.

[19] Cartin-Ceba R, Kashiouris M, Plataki M, Kor DJ, Gajic O, Casey ET (2012). Risk factors for development of acute kidney injury in critically ill patients: a systematic review and meta-analysis of observational studies. Crit Care Res Pract.

[20] Kane-Gill SL, Sileanu FE, Murugan R, Trietley GS, Handler SM, Kellum JA (2015). Risk factors for acute kidney injury in older adults with critical illness: a retrospective cohort study. Am J Kidney Dis.

[21] Levey AS, Stevens LA, Schmid CH (2009). A new equation to estimate glomerular filtration rate. Ann Intern Med.

[22] WHO. World Health Organization: Haemoglobin concentrations for the diagnosis of anaemia and assessment of severity. http://www.who.int/vmnis/indicators/haemoglobin.pdf (2011). Accessed 5 April 2016.

[23] Sutherland SM, Zappitelli M, Alexander SR (2010). Fluid overload and mortality in children receiving continuous renal replacement therapy: the prospective pediatric continuous renal replacement therapy registry. Am J Kidney Dis.

[24] Knaus WA, Wagner DP, Draper EA (1991). The APACHE III prognostic system. Risk prediction of hospital mortality for critically ill hospitalized adults. Chest.

[25] Hoste EA, Clermont G, Kersten A (2006). RIFLE criteria for acute kidney injury are associated with hospital mortality in critically ill patients: a cohort analysis. Crit Care.

[26] Zavada J, Hoste E, Cartin-Ceba R (2010). A comparison of three methods to estimate baseline creatinine for RIFLE classification. Nephrol Dial Transpl.

[27] DaL Hosmer S (2000). Applied logistic regression.

[28] Hoste EA, Bagshaw SM, Bellomo R (2015). Epidemiology of acute kidney injury in critically ill patients: the multinational AKI-EPI study. Intensive Care Med.

[29] Whelton A (1999). Nephrotoxicity of nonsteroidal anti-inflammatory drugs: physiologic foundations and clinical implications. Am J Med.

[30] Mann JF, Goerig M, Brune K, Luft FC (1993). Ibuprofen as an over-the-counter drug: is there a risk for renal injury?. Clin Nephrol.

[31] Musu M, Finco G, Antonucci R (2011). Acute nephrotoxicity of NSAID from the foetus to the adult. Eur Rev Med Pharmacol Sci.

[32] Uchino S, Bellomo R, Morimatsu H (2005). External validation of severity scoring systems for acute renal failure using a multinational database. Crit Care Med.

